# Combined Assessment of Diffusion Parameters and Cerebral Blood Flow Within Basal Ganglia in Early Parkinson’s Disease

**DOI:** 10.3389/fnagi.2019.00134

**Published:** 2019-06-04

**Authors:** Laura Pelizzari, Maria M. Laganà, Sonia Di Tella, Federica Rossetto, Niels Bergsland, Raffaello Nemni, Mario Clerici, Francesca Baglio

**Affiliations:** ^1^IRCCS, Fondazione Don Carlo Gnocchi, Milan, Italy; ^2^Department of Neurology, Buffalo Neuroimaging Analysis Center, Jacobs School of Medicine and Biomedical Sciences, University at Buffalo, State University of New York, Buffalo, NY, United States; ^3^Department of Pathophysiology and Transplantation, Università degli Studi di Milano, Milan, Italy

**Keywords:** DTI, ASL, diffusion parameters, cerebral blood flow, Parkinson’s disease

## Abstract

Diffusion tensor imaging (DTI) is a sensitive tool for detecting brain tissue microstructural alterations in Parkinson’s disease (PD). Abnormal cerebral perfusion patterns have also been reported in PD patients using arterial spin labeling (ASL) MRI. In this study we aimed to perform a combined DTI and ASL assessment in PD patients within the basal ganglia, in order to test the relationship between microstructural and perfusion alterations. Fifty-two subjects participated in this study. Specifically, 26 PD patients [mean age (SD) = 66.7 (8.9) years, 21 males, median (IQR) Modified Hoehn and Yahr = 1.5 (1–1.6)] and twenty-six healthy controls [HC, mean age (SD) = 65.2 (7.5), 15 males] were scanned with 1.5T MRI. Fractional anisotropy (FA), mean diffusivity (MD), axial diffusivity (AD), radial diffusivity (RD) maps were derived from diffusion-weighted images, while cerebral blood flow (CBF) maps were computed from ASL data. After registration to Montreal Neurological Institute standard space, FA, MD, AD, RD and CBF median values were extracted within specific regions of interest: substantia nigra, caudate, putamen, globus pallidus, thalamus, red nucleus and subthalamic nucleus. DTI measures and CBF were compared between the two groups. The relationship between diffusion parameters and CBF was tested with Spearman’s correlations. False discovery rate (FDR)-corrected *p*-values lower than 0.05 were considered significant, while uncorrected *p*-values <0.05 were considered a trend. No significant FA, MD and RD differences were observed. AD was significantly increased in PD patients compared with HC in the putamen (*p* = 0.005, p_FDR_ = 0.035). No significant CBF differences were found between PD patients and HC. Diffusion parameters were not significantly correlated with CBF in the HC group, while a significant correlation emerged for PD patients in the caudate nucleus, for all DTI measures (with FA: *r* = 0.543, p_FDR_ = 0.028; with MD: *r* = −0.661, p_FDR_ = 0.002; with AD: *r* = −0.628, p_FDR_ = 0.007; with RD: *r* = −0.635, p_FDR_ = 0.003). This study showed that DTI is a more sensitive technique than ASL to detect alterations in the basal ganglia in the early phase of PD. Our results suggest that, although DTI and ASL convey different information, a relationship between microstructural integrity and perfusion changes in the caudate may be present.

## Introduction

Parkinson’s disease (PD) is a progressive neurodegenerative disease that is characterized by early dopaminergic neuron loss in the substantia nigra pars compacta, leading to dopamine deficiency in the basal ganglia, and resulting in movement disorders (Kalia and Lang, 2015). Although non-motor symptoms such as sleep disorders, depression and cognitive impairment may be present, resting tremor, bradykinesia and rigidity are the hallmarks of the disease (Kalia and Lang, 2015; Obeso et al., 2017).

Parkinson’s disease diagnosis still remains largely clinical, based on the three cardinal symptoms onset, (Postuma et al., 2015) while dopamine transporter (DaT) single photon emission computerized tomography is currently used to confirm the clinical diagnosis (Seifert and Wiener, 2013). Nevertheless, the search for biomarkers with magnetic resonance imaging (MRI) is an area of active research in the field of PD. A number of MRI-based methods have been proposed to provide sensitive and non-invasive quantitative biomarkers of neurodegeneration (Obeso et al., 2017). In this framework, diffusion tensor imaging (DTI) and arterial spin labeling (ASL) are advanced MRI techniques that specifically allow for tissue integrity assessment (Alexander et al., 2007) and cerebral blood flow (CBF) quantification, respectively (van Osch et al., 2018).

Parameters derived from DTI, such as fractional anisotropy (FA) and mean diffusivity (MD), are suitable for investigating microstructural changes in the brain. MD provides a measure of overall diffusivity, while FA quantifies the extent to which diffusion is characterized by a preferential orientation. In addition to FA and MD, axial diffusivity (AD) and radial diffusivity (RD) can also be computed from DTI data. AD represents the primary eigenvalue describing water diffusivity while RD is determined by the average of the two smaller eigenvalues. Alterations of FA, MD, AD, and RD in white matter (WM), cortical and subcortical gray matter (GM) have been previously reported in PD patients, mirroring neurodegeneration and possible brain reorganization due to the disease (Atkinson-Clement et al., 2017). Furthermore, DTI measures in the subcortical areas were shown to be sensitive makers of disease progression in PD, (Cochrane and Ebmeier, 2013; Scherfler et al., 2013; Wei et al., 2016; Atkinson-Clement et al., 2017) even at the early stages of the disease (Taylor et al., 2018). DTI changes in the substantia nigra of PD patients were observed to be associated with increasing dopaminergic deficits, reduced α-synuclein and total tau protein concentrations in cerebrospinal fluid, while diffusivity alterations in the thalamus were correlated with cognitive decline in PD (Zhang et al., 2016).

Together with neuronal degeneration, metabolic and perfusion parameters may also be altered in PD due to either neurovascular unit function changes or increased cerebrovascular disease burden associated with aging (Al-Bachari et al., 2014). Given the value of CBF as a biomarker in PD, ASL MRI is a promising technique for PD assessment since it allows absolute CBF assessment without using an exogenous contrast agent (Pyatigorskaya et al., 2014). Indeed, abnormal cerebral perfusion patterns in PD have been revealed using ASL (Melzer et al., 2011). In addition, this technique proved to be effective in detecting CBF alterations in non-demented PD patients (Syrimi et al., 2017) and arterial transit time changes in idiopathic PD (Al-Bachari et al., 2014).

The combined assessment of FA and CBF has recently been proposed as an effective method for investigating pathological changes in the early stages of PD (Wei et al., 2016). Decreased FA in the substantia nigra and reduced CBF in the basal ganglia were reported in the same group of patients, hinting that different neuro-pathological processes may underlie the degeneration in the subcortical regions primarily involved in the disease (Wei et al., 2016).

To the best of our knowledge, MD, AD, and RD have not been assessed together with CBF as of yet. Furthermore, a correlation analysis between DTI and ASL-derived parameters is still missing in PD. Therefore, in this study we aimed to perform a combined DTI and ASL assessment in early PD patients to investigate FA, MD, AD, RD, and CBF alterations in the basal ganglia regions with respect to healthy controls (HC). In addition, we aimed to evaluate the correlation between microstructural and perfusion parameters. Due to the neurovascular coupling, a potential link between them was expected.

## Materials and Methods

### Subjects

Fifty-two subjects (26 PD patients and 26 HC) were included in this study. PD patients were consecutively recruited from the Neurorehabilitation Unit of the IRCCS Fondazione Don Gnocchi in Milan, while HC were enrolled between hospital personnel and volunteers. Only probable PD patients diagnosed according to the Movement Disorder Society Clinical Diagnostic Criteria for PD (Postuma et al., 2015) and with positive DaT scan were included in the study. Other inclusion criteria for PD group were: mild to moderate stages of the disease (Modified Hoehn and Yahr – H&Y<3), (Postuma et al., 2015) stable drug therapy with either L-Dopa or dopamine agonists, freezing assessed with Movement Disorder Society-sponsored revision of the Unified Parkinson’s Disease Rating Scale (MDS-UPDRS) part II lower than 2, time spent with dyskinesias assessed with MDS-UPDRS part IV lower than 2. Left-handed subjects, people with history of psychiatric disorders, neurological diseases other than PD, cardiovascular and/or metabolic diseases were excluded from the study. All the enrolled PD patients were clinically evaluated by an experienced neurologist within 2 weeks of the MRI scan. Specifically, the H&Y Scale and the MDS-UPDRS were used to assess the severity of PD symptoms (Goetz et al., 2004). Levodopa equivalent daily dose (LEDD) was also calculated for each PD patient (Tomlinson et al., 2010). Montreal Cognitive Assessment (MoCA) was used to evaluate the cognitive status of all recruited subjects to exclude frank dementia. For PD patients, additional cognitive assessments included the Trail Making Test (TMT), phonemic fluency and semantic fluency.

The study was approved by the IRCCS Fondazione Don Carlo Gnocchi Ethics Committee and performed in accordance with the principles of the Helsinki Declaration. Written and informed consent was obtained from all the participants.

### MRI Acquisition

All the enrolled subjects were scanned on a 1.5T Siemens Magnetom Avanto scanner, equipped with a 12-channel head coil. The MRI protocol included:

- Structural sequences:
  – A dual-echo turbo spin echo proton density PD/T2-weighted image [repetition time (TR) = 5550 ms, echo time (TE) = 23/103 ms, matrix size = 320 × 320 × 45, resolution 0.8 × 0.8 × 3 mm^3^] to exclude the presence of WM hyperintensities beyond those expected as a part of normal aging;
  – A 3D high-resolution T1-weighted image obtained with a magnetization-prepared rapid acquisition with gradient echo (MPRAGE) sequence (TR = 1900 ms, TE = 3.37 ms, TI = 1100 ms, matrix size = 192 × 256 × 176, resolution 1 × 1 × 1 mm^3^) as anatomical reference, and to evaluate GM volume differences between groups;
  – A 2D T1-weighted anatomical image with 5 mm-thick axial slices (TR/TE = 393/12 ms, matrix size = 128 × 128 × 26, resolution = 1.7 × 1.7 × 5 mm^3^) as anatomical reference for CBF map registration;
– Diffusion-weighted echo planar images (EPI) along 64 directions (*b*-value = 1500 s/mm^2^) and 3 b0 images (2 with A-P, 1 with P-A phase encoding) with the same parameters (TR/TE = 7800/109 ms, matrix size = 102 × 102 × 46, resolution = 2.5 × 2.5 × 2.5 mm^3^);
– Multi-delay pseudo-continuous ASL (pCASL) sequence with background suppressed 3D gradient and spin echo (GRASE) readout (TR/TE = 3500/22.58 ms, labeling duration = 1500 ms, 5 post-labeling delays (PLD) = [700, 1200, 1700, 2200, 2700] ms, 12 pairs of tag/control images for each delay, matrix size = 64 × 64 × 32, resolution = 3.5 × 3.5 × 5 mm^3^, distance between the center of imaging slices and labeling plane of 90 mm; 3 M0 images acquired with TR = 5000 ms) (Wang et al., 2013).

### MRI Processing

A visual quality check was performed for all the acquired MRI data prior to any analysis. MRI data processing was performed with FMRIB’s Software Library (FSL^1^) unless otherwise specified.

To avoid voxel misclassification during GM, WM and cerebrospinal fluid (CSF) automated segmentation, age-associated WM hyperintensities were identified (if any) on PD/T2-weighted images by an experienced neuroradiologist. Hyperintensities were segmented with Jim software, version 6.0^2^, and the obtained masks were registered to corresponding MPRAGE images with Advanced Normalization Tools (ANTs^3^) in order to perform lesion filling. Non-brain tissue was removed from lesion-filled MPRAGE image, then brain tissue classification was performed with SIENAX (Smith et al., 2002).

For processing of the diffusion-weighted images, the susceptibility-induced off-resonance field was estimated with the topup tool (Andersson et al., 2003). The eddy tool was then used to simultaneously correct images for eddy currents and subject movement as well as susceptibility-induced geometric distortions (Andersson and Sotiropoulos, 2016). Diffusion tensor estimation for each voxel was performed with dtifit (Behrens et al., 2003) and FA maps were derived. Each FA map was registered to the Montreal Neurological Institute (MNI) FA template with non-linear transformation, and the tensor was warped accordingly. Then, MD, AD, and RD maps were derived.

ASL raw data were corrected for movement with ANTs, then tag images were subtracted from control ones. CBF maps were calculated with the oxford_asl tool (Chappell et al., 2009) (tissue T1 = 1.2 s, T1 of blood = 1.36 s, tagging efficiency = 0.8) (Wang et al., 2013; Laganà et al., 2018) and calibrated with the asl_calib tool (Chappell et al., 2009) by adjusting for CSF magnetization extracted from M0 images. Partial volume effect (PVE) correction was performed based on the assumption that CBF in the GM is 2.5 times greater than in the WM (Marshall et al., 2016). Finally, GM CBF maps were registered to MNI standard space. To do this, PVE-corrected CBF maps were first linearly registered to the respective 2D T1-weighted images, characterized by the same slice thickness of ASL data, using ANTs. Then, non-linear registration to MNI standard space was performed via the MPRAGE with ANTs.

For each subject, median values of CBF, FA, MD, AD, and RD were extracted within specific regions of interest (ROIs). Specifically, median CBF, FA, MD, AD, and RD values were computed across the voxels in each ROI, namely caudate, putamen, globus pallidus, thalamus, substantia nigra, red nucleus and subthalamic nucleus. The Harvard-Oxford atlas was used to generate caudate, putamen, globus pallidus, and thalamus masks. Substantia nigra, red nucleus and subthalamic nuclei were defined from the Multi-contrast PD25 atlas (Xiao et al., 2015) and registered to MNI standard space. All the ROIs were eroded with a gaussian kernel (sigma = 2 mm) before performing the extraction of the median values of the parameters of interest.

To check for potential GM volume differences between PD patients and HC within the basal ganglia, voxel-based morphometry (VBM) was performed. This analysis was used to exclude that potential differences in diffusion parameters could be due to GM atrophy. Specifically, each subject’s GM map was non-linearly registered to MNI standard space, modulated with the Jacobian of the warp field and smoothed with a Gaussian kernel (sigma = 3 mm). GM volume voxel-wise comparison between the two groups was performed with the randomize tool, (Winkler et al., 2014) correcting for age and sex (ANCOVA), and using threshold-free cluster enhancement for cluster detection with 5000 permutations. The analysis was restricted to the basal ganglia regions of interest (i.e., substantia nigra, caudate, globus pallidus, putamen, thalamus, red nucleus and subthalamic nucleus). VBM results were family wise error (FWE) corrected at *p* < 0.05 to account for multiple comparisons.

### Statistical Analysis

Normality of data distributions was tested with the Shapiro-Wilk test and parametric or non-parametric statistics were used accordingly.

Age and sex differences between PD and HC group were tested with an independent samples *t*-test and Chi-squared test, as appropriate.

Since L-Dopa or dopamine agonists may have an impact on CBF, (Chen et al., 2015; Lin et al., 2016) LEDD and CBF were non-parametrically correlated (Spearman’s) with one another for all ROIs. In case of significant bivariate correlation, LEDD was included as a covariate.

The Mann-Whitney *U*-test was used to compare FA, MD, AD, RD and CBF measures between PD patients and HC. The relationship between each diffusion parameter and CBF was tested with Spearman’s correlations, in PD and HC group separately. The Benjamini-Hochberg procedure was performed to control for the false discovery rate (FDR). FDR-corrected *p*-values lower that 0.05 were considered significant. Uncorrected *p*-values lower than 0.05 were considered as trends. Eta squared was computed to estimate the effect size and subsequently transformed to Cohen’s d values. Effect size was classified as very small for d < 0.2, small for 0.2 ≤ d<0.5, moderate for 0.5 ≤ d<0.8 and large for d ≥ 0.8.

Additional analyses to assess the relationship between diffusion and perfusion alterations and disease duration were performed and are described in Supplementary Material (see Supplementary Table 1). In addition, correlation analysis between FA, MD, AD, RD, and CBF and the neuropsychological test scores were performed and are shown in Supplementary Material (see Supplementary Table 2).

## Results

### Demographics

PD and HC groups were age- and sex-matched (*p* = 0.527 and *p* = 0.071, respectively). PD patients and HC had a mean (standard deviation-SD) age of 66.7 (8.9) and 65.2 (7.5) years old, respectively. The PD group was characterized by a median (interquartile range-IQR) H&Y of 1.5 (1–1.6), and by a mean (SD) MDS-UPDRS III of 19.2 (11.2). The median adjusted MoCA (Santangelo et al., 2015) of the PD group and HC group was 24.3 and 25.6, respectively. Demographic and clinical characteristics of the two groups are reported in Table 1.

**Table 1:** Demographic and clinical characteristics of the participants of the study.

	HC (*n* = 26)	PD (*n* = 26)	*p*-value
Males, n (%)	15 (57.7)	21 (80.8)	0.071^a^
Age in yrs, mean (SD)	65.2 (7.5)	66.7 (8.9)	0.527^b^
H&Y, median (IQR)	na	1.5 (1–1.6)	–
MDS-UPDRS III score during on periods, mean (SD)	na	19.2 (11.2)	–
Disease duration in yrs, median (IQR)	–	3 (2–4)	–
Onset laterality, left n (%)	–	10 (38.5)	–
Antiparkinsonian medications			
Levodopa-containing drugs, n (%)	–	11 (42.3)	–
Dopamine agonists, n (%)		11 (42.3)	
MAO-B inhibitors, n (%)		17 (65.4)	
LEDD, mean (SD)	–	214.2 (121.5)	–
Education level in yrs, median (IQR)	16 (13-18)	13 (8–17.3)	0.060^c^
MoCA, median (IQR)	25.6 (24.2-27.7)	24.3 (21.6–26.3)	**0.042**^c^
TMT, Part A, median (IQR)	na	41.5 (30.3–63.5)	–
TMT, Part B, median (IQR)	na	73.5 (58.3–110.5)	–
TMT, B-A, median (IQR)	na	40.0 (25.0–63.8)	–
Phonemic fluency, mean (SD)	na	32.7 (9.6)	–
Semantic fluency, mean (SD)	na	39.7 (10.4)	–

*HC, healthy controls; IQR, interquartile range; LEDD, Levodopa daily dose equivalent; MAO-B, monoamine oxidase-B; Movement Disorder Society-sponsored revision of the Unified Parkinson’s Disease Rating Scale (MDS-UPDRS), MoCA, Montreal Cognitive Assessment, n, number, na, not available, PD, Parkinson’s disease, SD, standard deviation, TMT, Trail Making Test, yrs, years. MoCA scores were adjusted according to Santangelo et al. (2015), TMT according to Giovagnoli et al. (1996), phonemic fluency according to Carlesimo et al. (1996) and semantic fluency according to Novelli et al. (1986). Chi-squared test (a), independent samples Student’s t-test (b), and Mann-Whitney test (c) were used to evaluate differences between PD and HC groups, as appropriate. P-values lower than 0.05 were considered significant (in bold).*

### MRI Parameters Group Comparison

All the MRI images were classified as good quality scans and included in the analysis.

No significant correlation was found between CBF and LEDD in any ROI (results not shown). For this reason, LEDD was not considered as covariate in the following analysis.

No significant FA differences were observed. MD was significantly higher in PD patients with respect to HC in the substantia nigra (*p* = 0.030, *d* = 0.631), putamen (*p* = 0.012, *d* = 0.742) and red nucleus (*p* = 0.036, *d* = 0.607) but none of the results survived FDR correction. AD was found to be significantly higher in PD compared to HC in the putamen (*p* = 0.005, *d* = 0.836), even when correcting for multiple comparisons (p_FDR_ = 0.035). Increased RD was observed in PD for the putamen (*p* = 0.039, *d* = 0.596) and red nucleus (*p* = 0.034, *d* = 0.616), while higher CBF was found in the subthalamic nucleus (*p* = 0.022, *d* = 0.669) although significance was lost after FDR correction. Median FA, MD, AD, RD, and CBF values, and their IQR are reported in Table 2.

**Table 2:** Median (IQR) values of FA, MD, AD, RD, and CBF for HC and PD groups within the ROIs.

Parameter	ROI	HC (*n* = 26)	PD (*n* = 26)	PD vs. HC		
				p	p_FDR_	d
FA	Substantia Nigra	0.436 (0.411–0.463)	0.443 (0.422–0.457)	0.570	0.884	0.158
	Caudate	0.122 (0.114–0.136)	0.120 (0.109–0.141)	0.714	0.884	0.102
	Pallidum	0.241 (0.229–0.247)	0.237 (0.225–0.247)	0.884	0.884	0.041
	Putamen	0.140 (0.131–0.149)	0.139 (0.131–0.147)	0.840	0.884	0.056
	Thalamus	0.260 (0.250–0.271)	0.253 (0.244–0.261)	0.055	0.385	0.553
	Red Nucleus	0.372 (0.333–0.392)	0.355 (0.326–0.374)	0.305	0.712	0.287
	Subthalamic nucleus	0.437 (0.422–0.461)	0.434 (0.406–0.449)	0.257	0.712	0.319
MD	Substantia Nigra	0.725 (0.698–0.759)	0.750 (0.731–0.775)	**0.030**	0.084	0.631
[10^-3^ mm^2^/sec]	Caudate	0.726 (0.706–0.850)	0.766 (0.715–1.004)	0.227	0.281	0.340
	Pallidum	0.736 (0.707–0.759)	0.743 (0.717–0.780)	0.464	0.464	0.204
	Putamen	0.685 (0.675–0.704)	0.704 (0.694–0.718)	**0.012**	0.084	0.742
	Thalamus	0.733 (0.725–0.763)	0.754 (0.739–0.770)	0.073	0.128	0.514
	Red nucleus	0.620 (0.595–0.647)	0.646 (0.621–0.658)	**0.036**	0.084	0.607
	Subthalamic nucleus	0.666 (0.651–0.690)	0.677 (0.660–0.701)	0.241	0.281	0.329
AD	Substantia Nigra	1.117 (1.064–1.172)	1.147 (1.088–1.195)	0.188	0.373	0.372
[10^-3^ mm^2^/sec]	Caudate	0.836 (0.810–0.957)	0.874 (0.831–1.105)	0.162	0.373	0.396
	Pallidum	0.919 (0.891–0.977)	0.921 (0.890–0.994)	0.721	0.721	0.099
	Putamen	0.783 (0.769–0.802)	0.807 (0.792–0.825)	**0.005**	**0.035**	0.836
	Thalamus	0.947 (0.935–0.996)	0.968 (0.949–0.995)	0.213	0.373	0.350
	Red Nucleus	0.868 (0.830–0.914)	0.807 (0.792–0.825)	0.375	0.525	0.248
	Subthalamic Nucleus	0.995 (0.973–1.022)	0.987 (0.951–1.021)	0.674	0.721	0.117
RD	Substantia Nigra	0.543 (0.498–0.563)	0.559 (0.538–0.579)	0.082	0.134	0.497
[10^-3^ mm^2^/sec]	Caudate	0.682 (0.656–0.801)	0.719 (0.662–0.957)	0.241	0.281	0.329
	Pallidum	0.650 (0.623–0.670)	0.663 (0.631–0.691)	0.365	0.365	0.253
	Putamen	0.637 (0.627–0.655)	0.653 (0.635–0.667)	**0.039**	0.124	0.596
	Thalamus	0.633 (0.625–0.660)	0.657 (0.639–0.674)	0.053	0.124	0.556
	Red nucleus	0.481 (0.470–0.527)	0.519 (0.500–0.530)	**0.034**	0.124	0.616
	Subthalamic nucleus	0.507 (0.488–0.537)	0.529 (0.495–0.547)	0.096	0.134	0.475
CBF	Substantia Nigra	26.86 (22.98–30.80)	24.86 (20.58–31.60)	0.634	0.840	0.132
[ml/min/100 g]	Caudate	21.63 (18.21–24.41)	20.75 (16.72–24.07)	0.608	0.840	0.142
	Pallidum	19.99 (18.21–24.43)	21.83 (19.65–24.84)	0.241	0.840	0.329
	Putamen	25.14 (21.54–29.18)	24.02 (21.50–28.47)	0.840	0.840	0.056
	Thalamus	25.92 (19.59–30.45)	23.44 (21.29–27.97)	0.687	0.840	0.112
	Red Nucleus	27.94 (23.39–33.87)	27.58 (23.47–32.28)	0.728	0.840	0.097
	Subthalamic Nucleus	22.65 (20.83–27.23)	28.56 (23.20–31.07)	**0.022**	0.154	0.669

*AD, axial diffusivity; CBF, cerebral blood flow; FA, fractional anisotropy; d, Cohen’s d; FDR, false discovery rate; HC, healthy controls; IQR, interquartile range; MD, mean diffusivity; n, number; PD, Parkinson’s disease; RD, radial diffusivity; ROI, region of interest. The group differences were tested with Mann-Whitney test. Original p-values, FDR-corrected *p*-values and Cohen’s d are reported. P-values lower than 0.05 were considered significant (in bold). Effect sizes were originally calculated as eta squared and subsequently transformed into Cohen’s d values. Effect size was considered very small for d < 0.2, small for 0.2 ≤d < 0.5, moderate for 0.5 ≤d < 0.8, and large for d ≥ 0.8. Note that obtained p-values lower than 0.05 are associated with moderate to large effect size.*

The VBM analysis did not show any significant GM volume differences between PD and HC groups within the basal ganglia regions.

### Correlations Between DTI Parameters and CBF

**FIGURE 1:**
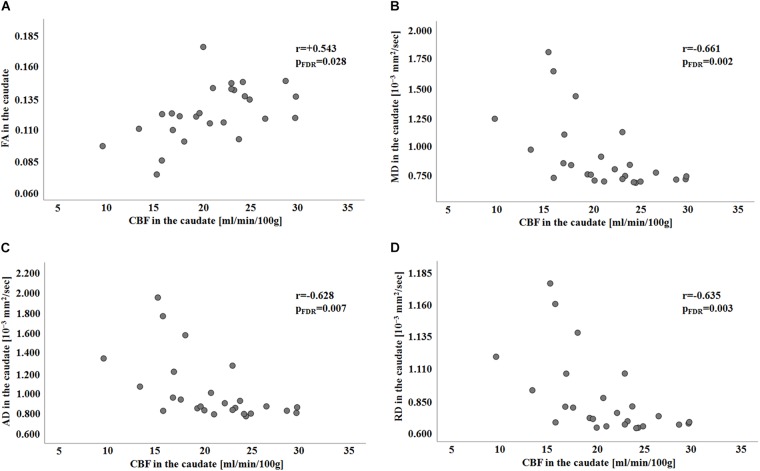
Scatterplots showing FA, MD, AD, and RD in the caudate in relation to CBF in the PD group (panel **A–D**, respectively). Spearman’s correlation coefficients and associated FDR-corrected *p*-values are shown (significant for p_FDR_ < 0.05). CBF, cerebral blood flow; FA, fractional anisotropy; FDR, false discovery rate; HC, healthy control; MD, mean diffusivity; AD, axial diffusivity; PD, Parkinson’s disease; r-Spearman’s correlation coefficient; RD, radial diffusivity.

No significant correlation was found between any of the diffusion parameters and CBF in HC group (Supplementary Table 3). Conversely, for the PD group, significant FDR-corrected correlations were found between CBF and all the diffusion parameters in the caudate nucleus (with FA: *r* = 0.543, p_FDR_ = 0.028; with MD: *r* = −0.661, p_FDR_ = 0.002; with AD: *r* = −0.628, p_FDR_ = 0.007; with RD: *r* = −0.635, p_FDR_ = 0.003; see Table 3). The scatterplots representing diffusion-vs.-perfusion measures in the caudate in the PD group are reported in Figure 1.

**Table 3:** Spearman’s correlation between local CBF and diffusion parameters in PD group (*n* = 26) within all the ROIs.

ROI	FA		MD		AD		RD	
	r	p_FDR_	r	p_FDR_	r	p_FDR_	r	p_FDR_
Substantia Nigra	+0.058	0.777	+0.015	0.941	−0.016	0.939	+0.201	0.692
Caudate	+0.543	**0.028**	−0.661	**0.002**	−0.628	**0.007**	−0.635	**0.003**
Pallidum	+0.382	0.126	−0.112	0.682	−0.032	0.939	−0.110	0.692
Putamen	−0.096	0.747	+0.320	0.391	+0.345	0.294	+0.0211	0.692
Thalamus	+0.128	0.747	−0.156	0.666	−0.175	0.688	−0.164	0.692
Red nucleus	−0.431	0.098	−0.236	0.572	−0.275	0.406	+0.024	0.906
Subthalamic Nucleus	+0.239	0.420	−0.284	0.666	−0.201	0.706	+0.123	0.692

*AD, axial diffusivity; CBF, cerebral blood flow; FA, fractional anisotropy; FDR, false discovery rate; MD, mean diffusivity; PD, Parkinson’s disease; RD, radial diffusivity; ROI, region of interest. P-values lower than 0.05 were considered significant (in bold).*

## Discussion

In the current study a combined assessment of DTI parameters and CBF within the basal ganglia was performed in a group of idiopathic PD patients to investigate the relationship between microstructural integrity and perfusion alterations. Three main findings were obtained. First, microstructural alteration was shown in the putamen, a region that is primarily involved in PD. In addition, no significant perfusion differences were observed between PD and HC in any of the considered ROIs. Finally, a significant correlation between DTI parameters and CBF emerged for PD patients in the caudate.

The presence of DTI alterations in PD has been extensively discussed over the last decade but without drawing final conclusions, as conflicting results have been reported (Cochrane and Ebmeier, 2013; Schwarz et al., 2013; Atkinson-Clement et al., 2017). Since the loss of dopaminergic neurons leads to the disruption of diffusion barriers, decreased FA and increased MD,AD and RD are expected in PD (Atkinson-Clement et al., 2017; Winklewski et al., 2018). Nevertheless, some recent studies also showed increased FA and decreased diffusivities in PD (Lenfeldt et al., 2015; Mole et al., 2016; Wen et al., 2016; Chen et al., 2018) In addition, varied multifocal patterns of abnormal DTI changes were reported, (Karagulle Kendi et al., 2008; Zhan et al., 2012) probably due to the multisystem involvement and the non-motor syndromes that characterize the disease (Hall et al., 2016). In the present study, significantly increased AD and a trend for higher MD and RD were found for PD patients in the putamen. The putamen is a key region for motor symptoms in PD, (Manza et al., 2016) since it is densely connected with the motor cortex. Therefore, its structural alterations are strongly associated with PD motor deficits (Nemmi et al., 2015) which are the cardinal symptoms of the disease. Notably, our PD group showed a trend for altered diffusivity also in the substantia nigra. Specifically, a trend for increased MD was observed, in line with several previous studies that reported altered nigral MD in PD (Scherfler et al., 2013; Schwarz et al., 2013; Du et al., 2014; Kamagata et al., 2016; Loane et al., 2016). Furthermore, a significant correlation between RD in the substantia nigra and TMT, part A score (Supplementary Table 2) was found. Conversely, no significant FA alterations were detected within the substantia nigra of PD patients in this study. Although this result is in contrast with several previous studies that reported reduced FA in PD, (Yoshikawa et al., 2004; Chan et al., 2007; Vaillancourt et al., 2009; Wei et al., 2016) heterogeneous FA alterations have been reported, so that FA in the substantia nigra has been considered insufficiently sensitive and specific to diagnose PD (Schuff et al., 2015; Hirata et al., 2017). The relatively limited sample size probably prevented us from consistently showing significant alterations of all the DTI parameters in the putamen and in the substantia nigra of our PD patients. However, the significantly altered AD in the putamen and the observed trends, associated with moderate to large effect sizes, suggest that DTI changes are present in the putamen and in the substantia nigra in early PD. The absence of group differences in terms of GM volumes within the regions showing DTI alterations highlighted that the loss of micro-structural integrity was without gross tissue loss (i.e., atrophy). Thus, the deafferentation of the nigrostriatal pathway likely induces a complex microstructural reorganization in the putamen and substantia nigra (Peran et al., 2010).

Besides DTI measures, ASL-derived CBF values were also tested in this study. No significant CBF differences between PD patients and HC were found within any of the ROIs. However, a trend for increased perfusion (significant before FDR-correction) was observed within the subthalamic nucleus. Hypermetabolism of the subthalamic nucleus, reflected by greater CBF, is in concordance with increased neuronal activity and an irregular firing pattern, as previously reported in PD (Hutchison et al., 1998; Blandini et al., 2000; Rodriguez-Oroz et al., 2001). The important role of the subthalamic nucleus in PD symptomatology and in direct-indirect pathway imbalance is supported by the dramatic clinical benefits experienced by PD patients after neurosurgery (both ablation and deep brain stimulation) targeting this structure (Obeso et al., 2017). Unlike in the DTI analysis, we did not detect any differences in putaminal perfusion in our PD patients. Our result of preserved CBF in the putamen is in line with some previous studies (Melzer et al., 2011; Al-Bachari et al., 2014; Pelizzari et al., 2019) but in contrast with a recent one that showed putaminal hypoperfusion in PD patients, both at early and middle stage of the disease (Wei et al., 2016). The considerable clinical heterogeneity that characterizes PD could have prevented us from identifying common patterns of CBF alterations in the basal ganglia in early PD patients. Investigating CBF in a wider cohort of PD patients at the early stage and accounting for motor symptom laterality onset is warranted to clarify the role of CBF changes in PD.

Interestingly, strongly significant correlations between all the DTI parameters and CBF were observed in the caudate nucleus of our PD patients, even though neither diffusion nor perfusion indices were altered. The caudate nucleus is known to be relatively spared at the early stage of the disease. A slower rate of dopaminergic decline in the caudate nucleus with respect to the putamen was reported by a previous study, with no significant changes in the caudate during the first years of the disease (Bruck et al., 2009). The dorsal caudate nucleus is connected with the dorsolateral prefrontal cortex, and it is part of the cognitive loop, which was proposed to be affected immediately after the motor one in PD (de la Fuente-Fernandez, 2012). Therefore, the absence of diffusion and perfusion changes in the caudate might be associated with the early disease stage. However, a correlation between DTI parameters and CBF was observed in this study. Specifically, PD patients who presented microstructural alterations in the caudate, showed also hypoperfusion. Both microstructural damage and perfusion alterations might be associated with disease duration (Supplementary Table 1), thus longitudinal studies are warranted to confirm their potential link with disease progression.

This study is not without limitations. The sample size was relatively small and the results remain to be confirmed in a larger number of patients. In addition, the relatively low resolution of ASL MRI, together with the small size of the ROIs, may have prevented us from showing the expected perfusion alterations. Another limitation that has to be mentioned is that the study was performed with a 1.5T MRI scanner. Although 3T scanners are characterized by a higher signal-to-noise ratio, the investigation of non-invasive markers to evaluate PD patients even with lower-field scanners, which are still prevalent in the clinical practice, is important in a translational perspective for diagnosis, treatment efficacy assessment and in terms of PD monitoring. The lack of a fine-grained neuropsychological assessment and the heterogeneity of our PD group in terms of laterality onset have also to be mentioned as limitations. This probably prevented us from showing consistent correlations between neuropsychological scores and MRI parameters (Supplementary Table 2). Finally, although we expected to find an association between perfusion changes and alterations of diffusion indices, only longitudinal studies may confirm the association with disease progression and allow for more firm conclusions to be drawn.

## Conclusion

In conclusion, DTI appears to be a more sensitive technique than ASL to detect changes in basal ganglia regions of early PD patients when using 1.5T clinical scanners. However, since CBF in the caudate correlates with respective DTI parameters, both microstructural alterations and hypoperfusion may potentially be involved in caudate neurodegeneration and in the development of further symptoms in later stages of the disease.

## Ethics Statement

The study was approved by the IRCCS Fondazione Don Carlo Gnocchi Ethics Committee and performed in accordance with the principles of the Helsinki Declaration. Written and informed consent was obtained from all the participants.

## Author Contributions

LP, ML, NB, MC, and FB contributed conception and design of the study. RN recruited PD patients. FB performed the clinical evaluation of PD patients. SDT and FR performed the neuropsychological evaluation of PD patients. LP performed the image processing and the statistical analysis and wrote the first draft of the manuscript. All authors contributed to manuscript revision, read and approved the submitted version.

## Conflict of Interest Statement

The authors declare that the research was conducted in the absence of any commercial or financial relationships that could be construed as a potential conflict of interest.

## Footnotes

1 http://www.fmrib.ox.ac.uk/fsl

2 http://www.xinapse.com/

3 http://stnava.github.io/ANTs
