# Supplementary material for: Combined Assessment of Diffusion Parameters and Cerebral Blood Flow Within Basal Ganglia in Early Parkinson’s Disease
Source: Front Aging Neurosci. 2019 Jun 4;11:134. doi: 10.3389/fnagi.2019.00134 (PMC6558180; doi:10.3389/fnagi.2019.00134)
Supplement: Supplementary file 1 [file Table_1.docx]

Supplementary Material

**Supplementary Table 1. Comparison of DTI parameters and CBF in the caudate between HC, PD patients that had disease duration shorter than 1 year and disease duration longer than 1 year.**

| **Parameter** | **PD dd≤1 yrs** | **PD dd>1 yrs** | **HC** | **Group comparison** | |
| --- | --- | --- | --- | --- | --- |
|  | **(N=5)** | **(N=21)** | **(N=26)** | **p** | **p_FDR_** |
| **FA** | 0.133 (0.111 -0.140) | 0.119 (0.109 -0.141) | 0.122 (0.114-0.136) | 0.697 | 0.697 |
| **MD** [10^−3^ mm^2^/sec] | 0.718 (0.690 -0.784) | 0.802 (0.729 -1.112) | 0.726 (0.706-0.850) | 0.073 | 0.172 |
| **AD [**10^−3^ mm^2^/sec] | 0.830 (0.790 -0.884) | 0.905 (0.845 -1.224) | 0.836 (0.810-0.957) | **0.044*** | 0.172 |
| **RD [**10^−3^ mm^2^/sec] | 0.662 (0.643 -0.740) | 0.751 (0.676 -1.054) | 0.682 (0.656-0.801) | 0.103 | 0.172 |
| **CBF** [ml/min/100g] | 23.60 (19.23 -24.48) | 19.91 (16.69 -23.56) | 21.63 (18.21-24.41) | 0.472 | 0.590 |

*** Post hoc analysis: PD(dd>1 yrs) > HC: p=0.045; PD(dd>1 yrs) > PD(dd≤1 yrs): p=0.041.**

**Legend:** CBF-cerebral blood flow, dd-disease duration, FA-fractional anisotropy, FDR- false discovery rate, MD-mean diffusivity, N-number, AD-axial diffusivity, RD-radial diffusivity, yrs-years.

PD patients (N=26) were split in two groups according to the disease duration (lower than 1 year- PD dd≤1 yrs, or longer than 1 year-PD dd>1 yrs). The group differences were tested with Kruskal-Wallis test. Post hoc analysis were performed with Mann-Whitney test. Both original p-values and FDR-corrected p-values are reported. FDR-corrected p-values lower than 0.05 were considered significant, while uncorrected p-values < 0.05 were considered a trend (in bold).

A trend for higher AD was observed in PD dd>1year with respect to PD dd≤1year and HC. In addition, PD dd>1year presented with lower median FA and CBF with respect to PD dd≤1year and HC, as well as higher median MD, AD, RD.

**Supplementary Table 2. Pearson’s partial correlations between local diffusion and perfusion parameters and the neuropsychological test scores in PD group (N=26).**

|  | **ROI** | **MoCA** | | **TMT, partA** | | **TMT, partB** | | **Phonemic fluency** | | **Semantic fluency** | |
| --- | --- | --- | --- | --- | --- | --- | --- | --- | --- | --- | --- |
|  |  | **r** | **p_FDR_** | **r** | **p_FDR_** | **r** | **p_FDR_** | **r** | **p_FDR_** | **r** | **p_FDR_** |
| **FA** | **Substantia Nigra** | +0.381 | 0.469 | -0.372 | 0.511 | -0.260 | 0.700 | +0.194 | 0.535 | +0.382 | 0.327 |
|  | **Caudate** | +0.033 | 0.910 | -0.104 | 0.732 | -0.081 | 0.706 | -0.187 | 0.535 | -0.135 | 0.620 |
|  | **Pallidum** | -0.024 | 0.910 | +0.146 | 0.732 | +0.124 | 0.700 | -0.288 | 0.401 | -0.171 | 0.595 |
|  | **Putamen** | +0.044 | 0.910 | +0.201 | 0.732 | +0.192 | 0.700 | -0.114 | 0.695 | -0.310 | 0.327 |
|  | **Thalamus** | +0.027 | 0.910 | +0.124 | 0.732 | +0.169 | 0.700 | +0.306 | 0.401 | +0.050 | 0.817 |
|  | **Red Nucleus** | +0.177 | 0.910 | -0.126 | 0.732 | -0.113 | 0.700 | +0.321 | 0.401 | +0.347 | 0.3267 |
|  | **Subthalamic Nucleus** | +0.122 | 0.910 | -0.060 | 0.780 | -0.161 | 0.700 | -0.012 | 0.956 | +0.204 | 0.5933 |
| **MD** | **Substantia Nigra** | -0.310 | 0.888 | +0.433 | 0.245 | +0.446 | 0.203 | +0.027 | 0.901 | -0.064 | 0.766 |
|  | **Caudate** | +0.060 | 0.888 | +0.190 | 0.960 | +0.133 | 0.910 | +0.237 | 0.767 | -0.079 | 0.766 |
|  | **Pallidum** | -0.074 | 0.888 | -0.103 | 0.960 | -0.167 | 0.910 | +0.091 | 0.785 | +0.166 | 0.765 |
|  | **Putamen** | -0.030 | 0.888 | -0.096 | 0.960 | -0.109 | 0.910 | +0.129 | 0.767 | +0.203 | 0.765 |
|  | **Thalamus** | +0.097 | 0.888 | -0.053 | 0.960 | -0.098 | 0.910 | +0.148 | 0.767 | +0.119 | 0.766 |
|  | **Red Nucleus** | -0.211 | 0.888 | -0.011 | 0.960 | +0.001 | 0.996 | +0.294 | 0.767 | +0.249 | 0.765 |
|  | **Subthalamic Nucleus** | -0.176 | 0.888 | -0.038 | 0.960 | +0.005 | 0.996 | +0.138 | 0.767 | +0.234 | 0.765 |
| **AD** | **Substantia Nigra** | -0.082 | 0.883 | +0.192 | 0.987 | +0.294 | 0.934 | +0.032 | 0.967 | +0.077 | 0.721 |
|  | **Caudate** | +0.059 | 0.883 | +0.195 | 0.987 | +0.134 | 0.934 | +0.229 | 0.936 | -0.092 | 0.721 |
|  | **Pallidum** | -0.090 | 0.883 | -0.091 | 0.987 | -0.186 | 0.934 | -0.009 | 0.967 | +0.084 | 0.721 |
|  | **Putamen** | +0.032 | 0.883 | -0.063 | 0.987 | -0.097 | 0.934 | +0.115 | 0.967 | +0.095 | 0.721 |
|  | **Thalamus** | +0.083 | 0.883 | +0.001 | 0.995 | 0.018 | 0.934 | +0.180 | 0.936 | +0.090 | 0.721 |
|  | **Red Nucleus** | -0.125 | 0.883 | +0.042 | 0.987 | -0.041 | 0.934 | +0.295 | 0.936 | +0.352 | 0.637 |
|  | **Subthalamic Nucleus** | -0.121 | 0.883 | -0.081 | 0.987 | -0.089 | 0.934 | +0.019 | 0.967 | +0.271 | 0.7 |
| **RD** | **Substantia Nigra** | -0.443 | 0.210 | +0.631 | **0.007** | +0.508 | 0.077 | -0.131 | 0.588 | -0.319 | 0.763 |
|  | **Caudate** | +0.052 | 0.867 | +0.194 | 0.667 | +0.137 | 0.655 | +0.235 | 0.588 | -0.078 | 0.8365 |
|  | **Pallidum** | -0.068 | 0.867 | -0.121 | 0.667 | -0.162 | 0.655 | +0.132 | 0.588 | +0.209 | 0.763 |
|  | **Putamen** | -0.036 | 0.867 | -0.115 | 0.667 | -0.118 | 0.655 | +0.138 | 0.588 | +0.241 | 0.763 |
|  | **Thalamus** | +0.096 | 0.867 | -0.105 | 0.667 | -0.166 | 0.655 | +0.116 | 0.588 | +0.132 | 0.8365 |
|  | **Red Nucleus** | -0.160 | 0.867 | -0.093 | 0.667 | -0.096 | 0.655 | +0.129 | 0.588 | +0.090 | 0.8365 |
|  | **Subthalamic Nucleus** | -0.371 | 0.259 | +0.191 | 0.667 | +0.251 | 0.655 | +0.154 | 0.588 | +0.018 | 0.934 |
| **CBF** | **Substantia Nigra** | +0.089 | 0.983 | +0.136 | 0.908 | -0.053 | 0.977 | -0.099 | 0.660 | -0.004 | 0.987 |
|  | **Caudate** | -0.032 | 0.983 | +0.111 | 0.908 | -0.031 | 0.977 | -0.245 | 0.660 | -0.113 | 0.987 |
|  | **Pallidum** | +0.237 | 0.983 | -0.095 | 0.9077 | -0.199 | 0.977 | +0.105 | 0.660 | +0.113 | 0.987 |
|  | **Putamen** | +0.139 | 0.983 | -0.070 | 0.9077 | -0.195 | 0.977 | -0.095 | 0.660 | +0.021 | 0.987 |
|  | **Thalamus** | -0.005 | 0.983 | +0.159 | 0.908 | -0.006 | 0.977 | -0.232 | 0.660 | -0.079 | 0.987 |
|  | **Red Nucleus** | +0.129 | 0.983 | +0.061 | 0.908 | -0.132 | 0.977 | -0.149 | 0.660 | -0.034 | 0.987 |
|  | **Subthalamic Nucleus** | +0.022 | 0.983 | 0.000 | 1.000 | -0.061 | 0.977 | +0.115 | 0.660 | +0.336 | 0.756 |

**Legend:** CBF-cerebral blood flow, FA-fractional anisotropy, FDR- false discovery rate, MD-mean diffusivity, N-number, AD-axial diffusivity, RD-radial diffusivity, yrs-years. Pearson’s partial correlations were performed by correcting for age and years of education. FDR-corrected p-values are reported. P-values lower than 0.05 were considered significant (in bold).

A significant positive correlation was observed between RD in the substantia nigra and TMT, part A score. Therefore, even if RD was not significantly altered in our PD patients, greater RD in the substantia nigra was associated with lower psychomotor speed.

**Supplementary Table 3. Spearman’s correlation between local CBF and diffusion parameters in HC group (n=26) within all the ROIs.**

| **ROI** | **FA** | | **MD** | | **AD** | | **RD** | |
| --- | --- | --- | --- | --- | --- | --- | --- | --- |
|  | **r** | **p_FDR_** | **r** | **p_FDR_** | **r** | **p_FDR_** | **r** | **p_FDR_** |
| **Substantia Nigra** | +0.353 | 0.166 | -0.357 | 0.259 | -0.171 | 0.682 | -0.361 | 0.245 |
| **Caudate** | +0.334 | 0.166 | -0.219 | 0.320 | -0.112 | 0.682 | -0.236 | 0.245 |
| **Pallidum** | +0.235 | 0.347 | -0.140 | 0.496 | -0.084 | 0.682 | 0.385 | 0.245 |
| **Putamen** | +0.137 | 0.588 | -0.215 | 0.340 | -0.287 | 0.546 | -0.249 | 0.245 |
| **Thalamus** | +0.429 | 0.123 | -0.237 | 0.340 | -0.103 | 0.682 | -0.269 | 0.245 |
| **Red Nucleus** | -0.028 | 0.893 | -0.408 | 0.259 | -0.337 | 0.546 | -0.325 | 0.245 |
| **Subthalamic Nucleus** | +0.416 | 0.123 | -0.295 | 0.320 | -0.230 | 0.602 | -0.254 | 0.245 |

**Legend:** FA-fractional anisotropy, MD-mean diffusivity, AD-axial diffusivity, RD-radial diffusivity, ROI-region of interest
